# Adherence with standard guidelines among doctors involved in aerosol generating procedures to control COVID-19 transmission

**DOI:** 10.12669/pjms.39.4.3971

**Published:** 2023

**Authors:** Madeeha Hassan, S H Waqar, Fatima Shahzad, Minhaj us Siraj

**Affiliations:** 1 Dr. Madeeha Hassan, MBBS. Postgraduate Resident, Department of Medicine, Pakistan Institute of Medical Sciences, Islamabad, Pakistan; 2 Dr. S H Waqar, MBBS, FCPS (Surgery), FICS, MCPS-HPE Professor and Head of Department of Surgery, Pakistan Institute of Medical Sciences, Islamabad, Pakistan; 3 Fatima Shahzad, Undergraduate Medical Student, Al-Nafees Medical College, Islamabad, Pakistan; 4 Dr. Minhaj us Siraj, MBBS, DOMS, MPH. Joint Executive Director, Pakistan Institute of Medical Sciences, Islamabad, Pakistan

**Keywords:** Aerosols, COVID-19, Guideline Adherence

## Abstract

**Objectives:**

To review the strategies for controlling COVID-19 transmission among doctors involved in aerosol generating procedures.

**Methods:**

A cross-sectional study was conducted in Pakistan Institute of Medical Sciences, Islamabad from September to November 2020, using a 21-itemed initially piloted self-administered questionnaire designed on Google forms. It was shared with doctors involved in Aerosol generating medical procedures (AGMPs) by using social media applications. After the data was collected, it was analyzed using SPSS version 21.

**Results:**

A total of 136 doctors participated in the study that included 59 female and 77 male participants. Our 67 (49.3%) participants screened their patients for signs and symptoms of COVID-19 before a routine checkup. The most common disinfectant used for reusable medical equipment by 62 (45.6%) respondents was alcohol swab, 57 (41.9%) did not disinfect reusable equipment and 17 (12.5%) used Dettol water spray.

**Conclusion:**

Majority of the doctors involved in AGMPS are not following the guidelines provided by international and national bodies. As such adherence to guidelines can help prevent COVID19 transmission in frontline doctors especially those involved in aerosol generating procedures.

## INTRODUCTION

COVID-19 is a highly contagious viral respiratory disease,^1^ primarily affecting lungs and airways, with signs and symptoms of fever, cough, shortness of breath and some extra-pulmonary symptoms like loss of sense of smell, diarrhea, nausea, vomiting and abdominal pain.^2^ It is potentially fatal if leads to Severe Acute Respiratory Syndrome (SARS).^3^ On December 31, 2019 in Wuhan city of China, the increasing cases of pneumonia of unknown cause were reported to World Health Organization China office, later on it was confirmed that the pathogen responsible is a virus of corona viridae family.^4^ WHO named this novel viral pneumonia as corona virus disease (COVID-19). International committee on taxonomy of viruses on the basis of its taxonomic and phylogenetic analysis named it as SARS-CoV-2.^5^ On January 13, 2020 first case was identified outside China, then spreading to the whole world.^6^ WHO declared it as a Public Health Emergency on Jan 30, 2020.^5^,^7^ With >118,000 confirmed cases in 114 countries, WHO declared COVID-19 a pandemic on March 11, 2020.^8^ Pakistan reported its first confirmed case of COVID-19 on February 26, 2020.^9^

COVID-19 is caused by SARS-CoV-2 that belongs to Corona family.^1^ Infection spreads through aerosols (respiratory droplets of < 5μm in diameter) reaching mucous membranes including eye, nose and mouth of nearby individuals making their way into the lungs.^3^ Virus shedding from infected person may also contaminate the environment containing both critical and non-critical medical equipment, however, main spread is through respiratory droplets rather than fomites.^10^

Health care workers are at a persistent occupational threat of developing COVID-19.^11^ Especially those working in mucous area specialities.^3^ An important source of nosocomial infection transmission in health care workers is Aerosol Generating Medical Procedures (AGMPs).^1^ AGMPs are the medical procedures that have the capability to generate aerosols excluding those which are normally formed during coughing, sneezing and talking.^1^ AGMPs may irritate the mucosa and cause retching or forceful cough generating aerosols.^1^ Aerosols are pathogen containing suspended air particles.^11^ A distance of at least one meter between patient and doctor is considered safe. However, it cannot be observed in case of medical procedures thus increasing the risk of contagion by many folds.^1^ Therefore, while working during a pandemic and being involved in high risk medical procedures, necessary precautions and use of PPEs is really crucial.^1^ Evidence suggests that there is a low risk of SARS-CoV-2 transmission while using PPEs.^11^ Adequate infection control is a must that includes minimizing number of HCWs in procedure room, most qualified person to perform the procedure and use of protective kits.^1^

Pakistan is ranked 28^th^ among the affected countries.^12^ In order to minimize the incidence the guidelines given by national and international authorities must be followed in health care facilities. This study observes the practical strategies being followed by the doctors involved in AGMPs.

## METHODS

A cross-sectional study was conducted in PIMS from September to November, 2020. The study participants were the doctors of Pakistan involved in Aerosol generating medical procedures. Ethical approval was taken from the Hospital Ethical Committee, Pakistan Institute of Medical Sciences (Ref. ECPIMS/14/2, Dated: September 8, 2020). Sample size of the study was calculated to be 136 using WHO sample size calculator keeping 95% confidence level and 7% margin of error. After a detailed literature review on international and national guidelines on performing aerosol generating procedures a 21-itemed self-made questionnaire was designed on Google forms as in this era of pandemic it was not advisable to collect the data manually. After successfully designing the questionnaire, a short pilot study was conducted on doctors involved in AGMPs. However, they were not included in the final analysis. After some minor changes the link was shared with Pakistani doctors involved in AGMPs by sending the link on social media applications. Convenience sampling was the sampling strategy. Doctors from different specialties including ENT, anaesthesia, dentistry, intensive care, pulmonology, medicine, surgery, paediatrics, neurosurgery and general duty, related to different types of health care facilities were contacted from different cities of Pakistan including Islamabad/Rawalpindi, Karachi, Faisalabad, Peshawar and Gujrat to take part in the study. Sign-in from email account was made mandatory in order to avoid data doubling from the respondents. Once the response of pre-decided sample size was achieved the option of accepting response was disabled. The data was copied from response sheet of Google docs and transferred to SPSS-21. Using SPSS-21 the data was analyzed and descriptive analysis was performed.

## RESULTS

A total of 136 doctors participated in the study that included 59 female and 77 male participants. Regarding the type of health care facility, 47 (34.6%) were working in surgical units, 25 (18.4%) in medical units, 15 (11%) in operation theatres, 15 (11%) in procedure rooms, 12 (8.8%) in OPDs and 10 (7.4%) in emergency rooms. The city of practice of 61 (44.9%) respondents was Islamabad/Rawalpindi, 32 (23.5%) were from Faisalabad, 28 (20.6%) from Karachi, nine (6.6%) from Peshawar and six (4.4%) from Gujrat. According to 67 (49.3%) participants, patients at their work place are screened for signs and symptoms of COVID-19 before a routine checkup.

If the practicing doctor came to know that patient might be a carrier of COVID-19, 77 (56.6%) participants referred the patient to infection control department, 39 (28.7%) deferred the patient for self-quarantine and 20 (14.7%) examined the patient. If the patient coming to respondents showed milder symptoms suggestive of COVID-19, 110 (80.9%) respondents referred the patient to infection control department, 21 (15.4%) examined the patient and four (2.9%) deferred the patients to self-quarantine for 14 days.

**Table-I:** shows the departments of participants involved in the study.

Doctors	Numbers	Percentage
ENT	26	19.1
Surgery	26	19.1
Anaesthesia	24	17.6
Pulmonology	20	14.7
Dentistry	18	13.2
Emergency Department	9	6.6
Gastroenterology	7	5.1
Intensive Care	3	2.2
Peadiatrics	1	0.7
Neurosurgery	1	0.7
General Medicine	1	0.7

**Fig.1:**
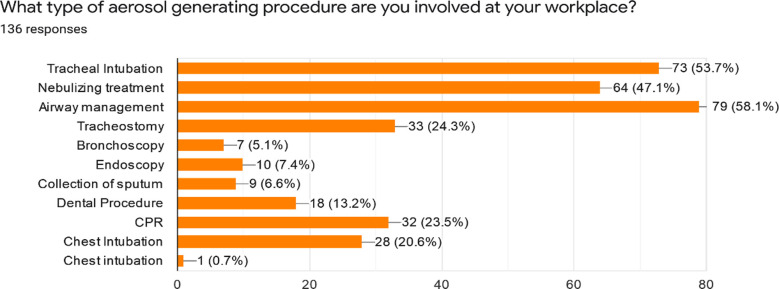
Aerosol Generating Procedures performed by the participants.

Participants involved in aerosol generating procedures that included tracheal intubation, nebulizing treatment, airway management, tracheostomy, bronchoscopy, sputum collection, dental procedure, CPR, chest intubation and Ultrasound guided Chest intubation are shown in Fig-1.

The disinfectant used for reusable dedicated medical equipment (BP cuffs, stethoscope, pulse dosimeter) by 62 (45.6%) respondents was alcohol swab, 57 (41.9%) did not disinfect reusable equipment and 17 (12.5%) used Dettol water spray.

According to our respondents the frequency of frequent hand contact surfaces decontamination was done after every procedure in 31 (22.8%), weekly in 26 (19.1%), twice daily in 23 (16.9%), thrice daily in 21 (15.4%), daily in 21 (15.4%), rarely in 12 (8.8%) and twice a week in two (1.5%) cases. For cleaning such surface’s wet mop was used in settings of 57 (41.9%) respondents, alcohol in 39 (28.7%), Dettol water in 15 (11%), detergent in nine (6.6%) and dry mop in four (2.9%).

The frequency of minimal hand contact surfaces decontamination was performed daily in 51 (37.5%), weekly in 46 (33.8%), after every procedure in 15 (11%), twice daily in 11 (8.1%), thrice daily in six (4.4%), twice weekly in five (3.7%) and rarely in two (1.5%) of cases. For cleaning such surfaces diluted phenyl water was used in 63 (46.3%), sodium hypochlorite in 28 (20.6%), alcohol based cleanser in 17 (12.5%), soap water in 15 (11%), detergent in seven (5.1%), hydrogen peroxide in four (2.9%) and diluted sodium hypochlorite in 2 (1.5%) settings.

## DISCUSSION

The burden of a recent pandemic on a third world country like Pakistan with incapacitated health system is held up by the health care workers. Due to scarcity of health care facilities, the already existing ones pose a great risk of contagion to HCWs, patients and their attendants due to overcrowding. Poor aeration of health care facility, prolonged exposure and non-compliance of SOPs make the matter worse. According to the American Society of Testing and Materials guidelines as discussed by Romano,^3^ patient visiting health care facility should be triaged first, screened for temperature and then should be sanitized with mandatory use of face mask and gloves and then only should be allowed to enter in the clinical facility. A short quick history can be taken from patients presenting in clinics or on elective surgical list, to determine their risk of contracting COVID-19.^13^ About 49.3% of respondents revealed that triage is done at their settings, rest of the respondents did not follow this trend. Roy Soetikno suggested that all patients presenting to Outpatient settings must be screened for symptoms.^14^ Those, who are suspected must be tested first.

UK guidelines^15^ suggest that unless the suspected/confirmed cases of COVID-19 are critically ill, they should self-isolate themselves for 14 days. In case of suspected COVID-19 patients, 28.7% of our respondents and for confirmed cases of COVID-19, 2.9% followed this guideline but majority referred the patients to infectious department. For a third world country like Pakistan where the health system is already incapacitated, a natural calamity like COVID-19 can only be dealt by using wise strategies. Referring suspected or mildly ill patients of COVID-19 to infectious ward might increase the work load of hospitals and will aid in contagion, rather, for such patient’s self-quarantine is recommended.

**Fig.2:**
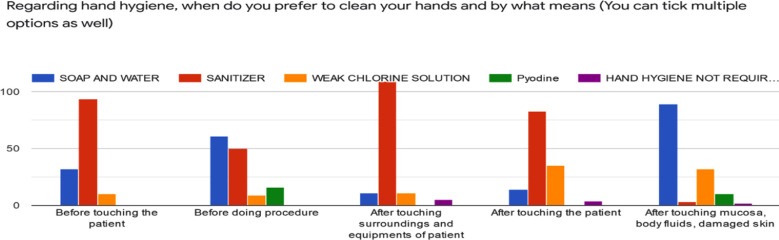
Cleansing agent used by our participants at five moments recommended by WHO.

The hand hygiene has been made essential by World Health Organization at five moments,^3^ where the use of alcohol based cleanser is recommended but soap and water use is superior to alcohol in minimizing viral load. However, according to our results (Fig.2), respondents use alcohol based sanitizer a lot more as compared to soap and water probably because it is less time consuming. Use of alcohol and soap with water are of equal importance,^16^ however, if hands are visibly contaminated soap with water is preferred over alcohol.^17^ Alcohol based cleansers must contain at least 60% of alcohol and skin should be exposed to alcohol for at least 20 to 30 seconds until dry.^1^

As for soap and water, the recommended duration of cleaning is 60 to 90 seconds. Local authorities for making infection control guidelines also make it clear that using hand gloves is not a substitute for hand hygiene. Hand hygiene is a must, where and when needed as recommended by World Health Organization.^18^

According to the guidelines set by NHRSC all the non-clinical reusable medical equipment should be decontaminated using alcohol with concentration of 60-90%. Our study depicts that respondents did not strictly follow these national guidelines as 41.9% of participants did not decontaminate these items.

For cleaning the surfaces regular use of 5% H_2_O_2_ or 60-70% alcohol or 0.1% sodium hypochlorite eliminates virus as they act against the lipid component of virus and destroys it.^16^, ^18^ If a reusable mop is used, it should be immersed in sodium hypochlorite for at least 2 minutes.^10^

National Institute of Health (NIH) Pakistan in its guidelines mentions chlorine based solutions as having more antimicrobial effect including both enveloped and non-enveloped viruses as compared to alcohol. More than two fifths i.e., 45.6% of our participants used alcohol to disinfect medical equipment. Some of the advantages of alcohol are that it is non-toxic, effective for surfaces or equipment, rapid bactericidal and low cost. This explains the trend of using alcohol by our respondents.^18^

Procedures with lower risk of contagion include airway placement, suctioning, CPR.^7^ Whereas, AGMPs like high flow nasal cannula, CPAP/BIPAP, bronchoscopy, GI endoscopy and endotracheal intubation are considered high risk procedures. A local study suggests use of appropriate PPE which includes long sleeved surgical gowns, hand gloves, goggles and an N95 or FFP2/P2 mask. Preferably a surgical mask and face shield over the respirator to increase its life.^13^ As per recommendations of Chinese Society of Anesthesiology use of N95 masks, shoe covers, surgical caps, gloves, surgical gowns, face shields and goggles are a must for intubation. Intubation should only be considered in patients with severe respiratory distress or hypoxemia.^7^

In addition to upper GI procedures, lower GI procedures are also considered as high risk but are not included in AGMPs. As along with the body secretions and aerosols, fomites and faeces (feco-oral route) can also transmit the infection,^14^,^19^ therefore, American Gastroenterological Association recommends use of N95 masks and double gloves while performing GI procedures regardless of the COVID-19 status of the patient.

**Fig.3:**
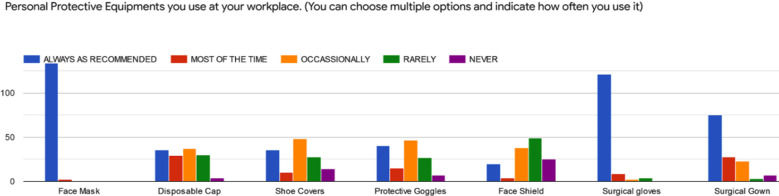
Use and frequency of different protective items by the respondents during the attempt of aerosol generating procedure.

For dentists, as eyes and nose are the most affected facial areas where aerosols produced during procedures are radiated^16^ therefore, use of N95 mask is recommended along with surgical gowns and goggles, especially for the ones who are involved in vigorous procedures.^19^ In a study in Pakistan 15% of dentists were reported to wear proper PPE including gloves, N95, goggles, head-cap, gown while performing AGMPs.^20^ However, use of goggles may hinder the accuracy of procedure, cause uneasiness and can impair vision.^19^ According to this study (Fig.3), the commonly used PPEs by the respondents are face masks, surgical gloves and surgical gowns.

Disinfection of procedure room or Operating theatres should be done at least thrice daily for minimum 30 minutes with disinfectant containing chlorine content in liquid.^21^ The guidelines of National Institute of Health Pakistan recommend strong chlorine solution with a concentration of 0.5% to disinfect surfaces and objects.^18^

### Limitation of the Study:

Limitations of our study include small sample size as data was collected from doctors of different specialties from only five cities of Pakistan that reduces the generalizability of the results. Regarding the use of PPEs, their reuse was not questioned at all whether these were reused and if reused, whether regularly decontaminated or not.

## CONCLUSION

Majority of the doctors involved in AGMPS are not following the guidelines provided by international and national bodies. As such adherence to guidelines can help prevent COVID19 transmission in frontline doctors especially those involved in aerosol generating procedures.

### Authors Contribution:

**MH:** Conceptualization of study, data collection, statistical analysis, manuscript writing, and accountable for the accuracy of study.

**SHW and MS:** Edited, reviewed and finally approved the manuscript.

**FS:** Performed data collection and participated in manuscript writing.
